# Antiviral Activities and Putative Identification of Compounds in Microbial Extracts from the Hawaiian Coastal Waters 

**DOI:** 10.3390/md10030521

**Published:** 2012-02-24

**Authors:** Jing Tong, Hank Trapido-Rosenthal, Jun Wang, Youwei Wang, Qing X. Li, Yuanan Lu

**Affiliations:** 1 Department of Public Health Sciences, University of Hawaii at Manoa, East-West Road, Honolulu, HI 96822, USA; Email: tongjing1975@yahoo.com.cn; 2 Key Laboratory of Combinatorial Biosynthesis and Drug Discovery (Wuhan University), Ministry of Education, and Institute of Traditional Chinese Medicine & Natural Products, School of Pharmaceutical Sciences, Wuhan University, Wuhan 430071, China; Email: wyw@wbgcas.cn; 3 Center for Marine Microbial Ecology and Diversity, University of Hawaii at Manoa, Honolulu, HI 96822, USA; Email: rosenthl@hawaii.edu; 4 Department of Molecular Biosciences and Bioengineering, University of Hawaii at Manoa, East-West Road, Honolulu, HI 96822, USA; Email: wangjun2@hawaii.edu

**Keywords:** marine extract, antiviral drug, antiviral activity, enveloped virus, secosteroids

## Abstract

Marine environments are a rich source of significant bioactive compounds. The Hawaiian archipelago, located in the middle of the Pacific Ocean, hosts diverse microorganisms, including many endemic species. Thirty-eight microbial extracts from Hawaiian coastal waters were evaluated for their antiviral activity against four mammalian viruses including herpes simplex virus type one (HSV-1), vesicular stomatitis virus (VSV), vaccinia virus and poliovirus type one (poliovirus-1) using *in vitro* cell culture assay. Nine of the 38 microbial crude extracts showed antiviral potencies and three of these nine microbial extracts exhibited significant activity against the enveloped viruses. A secosteroid, 5α(H),17α(H),(20*R*)-beta-acetoxyergost-8(14)-ene was putatively identified and confirmed to be the active compound in these marine microbial extracts. These results warrant future in-depth tests on the isolation of these active elements in order to explore and validate their antiviral potential as important therapeutic remedies.

## 1. Introduction

Viruses are known to cause a variety of infectious diseases which threaten public health. The search for new therapeutic agents to control virus infection is one of the highest priorities in virological research. Oceans represent a virtually untapped resource for the discovery of novel bioactive compounds [1]. The Census of Marine Life Project recently increased the estimate of marine species from more than 230,000 to at least one million marine organisms, and tens or even hundreds of millions of different microbes, such as protists, bacteria and archaea [2]. Secondary metabolites with various biological activities can be induced as a result of the complicated marine environment, some of which represent a valuable resource waiting to be discovered for the treatment of infectious diseases [3]. Hundreds of new compounds are reportedly identified from marine species each year [3], and several marine bioactive metabolites have been successfully developed by the pharmaceutical industry [4]. The Hawaiian archipelago, located in the middle of the Pacific Ocean, is the most isolated group of islands in the world [5]. Because of its unique geographical location, the Hawaiian tropical marine ecosystem promotes the generation of a higher percentage of endemic species relative to other tropical regions [6]. In this study, we have particularly tested some marine microbial extracts prepared from Hawaiian coastal waters to evaluate their antiviral activities.

Marine organisms have proven to produce many important therapeutic agents and are a continued focus for drug discovery [7]. The diversity of organisms in the marine environment has inspired researchers for many years to identify active compounds for clinical treatment of infectious diseases, including antifungal, antibacterial, antiprotozoal and antiviral activities. Each year hundreds of new bioactive compounds from marine environments are discovered and some have been approved as new therapeutic drugs by the Food and Drug Administration (FDA) and the European Agency for the Evaluation of Medicinal Products (EMEA). These include cytarabine (Cytosar-U^®^, Depocyt^®^), vidarabine (Vira-A^®^), ziconotide (Prialt^®^) and trabectedin (Yondelis^®^) [8]. 

To explore marine compounds for antiviral potential, more than 2,000 crude extracts from a variety of marine organisms, including sponges, bacteria and algae, have been prepared in our laboratories. A cell line bank has also been established, comprising more than 150 cell lines derived from various organs and tissues of different animal species. The objectives of this study were to (1) establish an *in vitro* model system to screen marine microbial extracts for their antiviral potential; (2) evaluate 38 marine crude extracts for their antiviral potency and (3) isolate and identify bioactive compounds in marine microbial crude extracts. Four representative viruses isolated from mammal species collected and prepared in the Environmental Health Laboratory at the University of Hawaii at Manoa were tested in this study (Table 1). 

**Table 1:** Four viruses used for the antiviral activity tests with the Vero host cells from the African Green Monkey kidney epithelial cells.

Viruses	Viral Family	Enveloped	Structure
Herpes simplex virus type one (HSV-1)	*Herpesviridae*	Yes	Linear double-stranded DNA genome
Vesicular stomatitis virus (VSV)	*Rhabdoviridae*	Yes	Single-stranded negative-sense RNA genome
Vaccinia virus	*Poxvirus*	Yes	Linear double-stranded DNA genome
Polio virus type one (poliovirus-1)	*Picornaviridae*	No	Single-stranded positive-sense RNA genome

## 2. Results and Discussion

### 2.1. Extract Cytotoxicity

**Figure 1:**
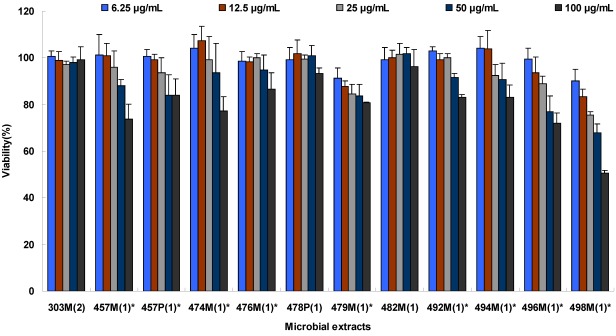
Cytotoxic sensitivities of Vero cells to selected marine microbial extracts. (^*^ Samples with significant differences among different concentrations in ANOVA tests. Vero cell at their exponential growth phase were seeded in 96-well plates and then exposed to different concentrations of selected marine extracts (4 wells per concentration). Following a 2-day incubation time at 37 °C, cell viability was determined using MTT assay by measuring absorbance reading at 492 nm. Results shown represent mean values of cell viability from two independent experiments and error bars denote the standard deviation.)

To assess properly the antiviral effect of the crude marine extracts, a set of experimental tests were performed to determine a safe and effective dose of these extracts to be used for antiviral experimental tests *in vitro*. 10% inhibition concentration (IC_10_) values were calculated by SPSS 16.0 [9]. Experimental results revealed that crude extracts 219P(3), 258M(1), 258P(1), 331P(3), 435P(1), 464P(1), 478M(1), 496P(1), 497P(1) were toxic to Vero cells with IC_10_ ≤ 5 µg/mL, and one extract 492P(1) was insoluble. These ten extracts were therefore not included in the antiviral assays. Extracts 62M(1), 62P(1), 226P(3), 298M(1), 298P(1), 397P(1), 456P(1), 457P(1), 457M(1), 460M(1), 474M(1), 476M(1), 479M(1), 485P(1), 485M(1), 492M(1), 494M(1), 495P(1), 496M(1) and 498M(1) showed variable levels of cytotoxicity with IC_10_ < 100 μg/mL (Figure 1 and Table 2). Based on the calculated IC_10_, subtoxic concentrations of the 28 viable extracts were used in the latter experimental tests. For extracts demonstrating no toxicity to Vero cells at all tested concentrations, the highest concentration of 100 μg/mL was selected for the following antiviral experimental tests.

**Table 2:** Marine extracts and their cytotoxicity ^a^.

Extracts ^b^	Sources	IC_10_ (μg/mL)
62M(1)	*Oscillatoria *sp*.*(Waikiki Aquarium)	64.3 ± 5.7
62P(1)	*Oscillatoria *sp*.*(Waikiki Aquarium)	49.2 ± 0.3
219P(3)	*Pseudoalteromonas *sp*. *from Mycale armata,Kaneohe Bay	≤5.0
226P(3)	*Pseudoalteromonas *sp*. *from Mycale armata,Kaneohe Bay	13.5 ± 1.1
258P(1)	*Oscillatoria *sp*.* from Fresh water	≤5.0
258P(1)	*Oscillatoria *sp*.* from Fresh water	≤5.0
298M(1)	bacterium from Kaneohe Bay, yet to be taxonomically classified	42.5 ± 1.5
298P(1)	bacterium from Kaneohe Bay; yet to be taxonomically classified	17.33 ± 0.17
303M(2)	bacterium from Kaneohe Bay; yet to be taxonomically classified	≥100
331P(3)	*Achromobacter fischeri *from *Mycale armata*, Kaneohe Bay	≤5.0
397M(1)	bacterium from Kaneohe Bay; yet to be taxonomically classified	≥100
397P(1)	bacterium from Kaneohe Bay; yet to be taxonomically classified	6.5 ± 0.4
435P(1)	bacterium from Hawaii Ocean Time Series (HOTS) site; yet to be taxonomically classified	≤5.0
456P(1)	Marine bacterium from HOTS site; yet to be taxonomically classified	9.1 ± 0.2
457M(1)	Diatom, *Amphora *sp., from Great Salt Lake	44.6 ± 1.9
457P(1)	Diatom, *Amphora *sp., from Great Salt Lake	51.7 ± 2.3
460M(1)	bacterium from HOTS site; yet to be taxonomically classified	22.5 ± 3.2
464P(1)	bacterium from HOTS site; yet to be taxonomically classified	≤5.0
474M(1)	bacterium; yet to be taxonomically classified	63.1 ± 4.2
475M(1)	bacterium; yet to be taxonomically classified	≥100
476M(1)	bacterium; yet to be taxonomically classified	48.9 ± 3.0
477P(1)	bacterium; yet to be taxonomically classified	≥100
478M(1)	bacterium; yet to be taxonomically classified	≤5.0
478P(1)	bacterium; yet to be taxonomically classified	≥100
479M(1)	bacterium; yet to be taxonomically classified	7.4 ± 0.5
482M(1)	bacterium; yet to be taxonomically classified	≥100
483P(1)	bacterium; yet to be taxonomically classified	≥100
485M(1)	bacterium; yet to be taxonomically classified	68.9 ± 5.8
485P(1)	bacterium; yet to be taxonomically classified	54.8 ± 4.7
492M(1)	bacterium; yet to be taxonomically classified	61.0 ± 3.2
492P(1)	bacterium; yet to be taxonomically classified	indissoluble
494M(1)	bacterium; yet to be taxonomically classified	44.0 ± 2.8
495M(1)	bacterium; yet to be taxonomically classified	≥100
495P(1)	bacterium; yet to be taxonomically classified	24.7 ± 1.8
496M(1)	bacterium; yet to be taxonomically classified	16.2 ± 1.3
496P(1)	bacterium; yet to be taxonomically classified	≤5.0
497P(1)	bacterium; yet to be taxonomically classified	≤5.0
498M(1)	bacterium; yet to be taxonomically classified	6.6 ± 0.6

^a^ Cytotoxicity is expressed as extracts concentration causing 10% inhibition of cell proliferation (IC_10_); ^b^ Each extract of marine microorganism was named, based on the following regulation. For example, extract 62M(1), where 62 denotes microorganism sequence number, cultured in the Center for Marine Microbial Ecology and Diversity and (1) denotes the first inoculation of this particular culture and subsequent inoculation of the same culture are labeled as (2), (3), *etc*. Cultures that had both the media/supernatant and pellet extracted are differentiated from one another by the addition of an M or P to the extracts sequence number to denote a media and pellet extraction. To harvest and extract marine bacteria, cultures were centrifuged at 5000 g for 20 min. The supernatant was then extracted with ethyl acetate and the sediment extracted with methylene chloride: 2-propanol (V/V, 2:1). The extracts samples were then dissolved in DMSO with a concentration of 100 mg/mL and then used for screening.

### 2.2. Viral Inhibition by Cell Pretreatment

Selected extracts were first tested for their anti-viral effect during the adsorption phase by blocking cellular receptor. Following 1 h pre-incubation with these extracts, cells were then infected with test viruses. Our data demonstrated that none of the marine extracts were able to prevent test viruses from entering Vero cells under the test conditions described. This indicated the marine extract treatments had no effect on viral infection and replication during the pretreatment phase. 

### 2.3. Viral Inhibition by Virus Pretreatment

**Figure 2:**
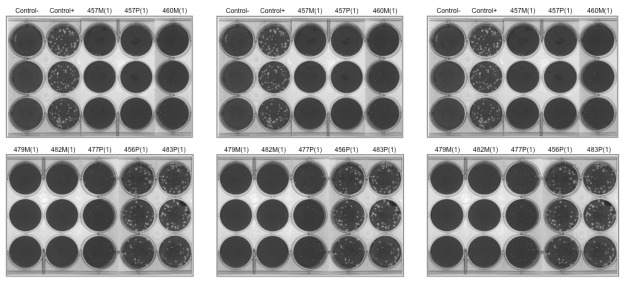
Marine microbial extracts mediated anti-viral attachment and entry into Vero cells. (HSV-1 (Left), VSV (middle), and Vaccinia virus (right) were pre-incubated with selected marine extracts at subtoxic concentration, incubated in room temperature for 2 h, and then used to infect Vero cells prepared in 24-well plates. Following 1 h viral adsorption, removed the viruses, then overlay medium was added and plates were transferred to 37 °C incubator for 36–48 h to allow viral plaque development. Plates were fixed and stained with crystal violet staining solution and photomicrographs were taken. Viral induced plaques were visually counted and marine extracts mediated inhibitory effect was determined by comparing the plaques produced in the control cultures.)

The marine extracts were then tested for their ability to combat viral infection by blocking viral entry into the cells or through viral inactivation. A total of 28 marine extracts were tested. None of the extracts exhibited any antiviral impact against poliovirus-1. However, most of the extracts exhibited antiviral effects against HSV-1, vaccinia and VSV at different levels based on viral plaque formation (Figure 2). As shown in Table 3, a total of 18 extracts showed antiviral effects against HSV-1 in Vero cells. In particular, 457M(1), 457P(1), 478P(1), 482M(1), 492M(1) and 494M(1) possessed strong antiviral activity, and a single treatment with one of these extracts resulted in complete deprivation of HSV-1 infectivity, leading to neither viral plaque formation nor viral-induced cytopathic effect (CPE) of the affected cells. Seven extracts including 226P(3), 460M(1), 475M(1), 476M(1), 485M(1), 485P(1), and 496M(1) exhibited moderate inhibitory effects on HSV-1 while the five other extracts, 303M(2), 474M(1), 477P(1), 479M(1) and 498M(1) showed slight inhibitory anti-HSV-1 effects.

**Table 3:** Marine microbial extracts and their antiviral effects in viral adsorption inhibition assay.

Extracts	Concentrations (μg/mL)	HSV-1	VSV	Vaccinia	Poliovirus-1
62M(1)	50	-	-	-	-
62P(1)	50	-	+++	-	-
226P(3)	12.5	++	-	-	-
298M(1)	50	-	+++	-	-
298P(1)	12.5	-	++	-	-
303M(2)	100	+	+++	+	-
397M(1)	100	-	-	-	-
397P(1)	6.25	-	-	-	-
456P(1)	12.5	-	-	-	-
457M(1)	25.0	+++	+++	+++	-
457P(1)	25.0	+++	+++	+++	-
460M(1)	25.0	++	++	-	-
474M(1)	50.0	+	+++	++	-
475M(1)	100	++	++	+	-
476M(1)	50.0	++	+++	++	-
477P(1)	100	+	+	++	-
478P(1)	100	+++	+	+++	-
479M(1)	6.25	+	+++	+++	-
482M(1)	100	+++	+++	+++	-
483P(1)	100	-	-	-	-
485M(1)	50.0	++	++	++	-
485P(1)	50.0	++	-	++	-
492M(1)	50.0	+++	++	++	-
494M(1)	50.0	+++	++	++	-
495M(1)	100	-	-	-	-
495P(1)	25.0	-	-	-	-
496M(1)	12.5	++	+++	++	-
498M(1)	6.25	+	+++	++	-

-: no meaningful inhibition at subtoxic concentration; +: Slight inhibition (≥20% and <50%); ++: Moderate inhibition (≥50% and <80%); +++: High inhibition (≥80%). 50–100 PFU/mL of virus diluted solution in serum-free medium and equal volumes of the extract dilutions were placed in a tube and mixtures incubated at room temperature for 1 h. Serum-free medium without extract was used as a blank control. The samples were then placed on monolayers of Vero cells to absorb for 1 h at 37 °C. The inhibition efficiency was assessed by counting plaques. Then the ratio of inhibition efficiency was calculated compared to the blank control.

As shown in Table 3 and Figure 2, the tested extracts also showed varied levels of antiviral impact against VSV. Eleven extracts including 62P(1), 298M(1), 303M(2), 457M(1), 457P(1), 474M(1), 476M(1) 479M(1), 482M(1), 496M(1) and 498M(1) showed strong antiviral activity, with 303M(2), 457P(1), 457M(1), 474M(1), 479M(1), 496M(1) and 498M(1) specifically resulting in complete viral inhibition evidenced by neither viral plaque formation nor virus-induced CPE appearance in Vero cells. In comparison, six extracts 298P(1), 460M(1), 475M(1), 485M(1), 492M(1) and 494M(1) exhibited a moderate antiviral effect and two extracts 477P(1) and 478P(1) showed slight viral inhibition while the remaining nine extracts showed no meaningful antiviral effect.

Similarly, these extracts also showed different antiviral effects on vaccinia virus as summarized in Table 3 and Figure 2. Among 28 marine extracts tested, extracts 457M(1), 457P(1), 478P(1), 479M(1) and 482M(1) showed strong antiviral activity, especially extracts 457P(1) and 457M(1) which completely blocked vaccinia infection in Vero cells. Nine other extracts 474M(1), 476M(1), 477P(1), 485M(1), 485P(1), 492M(1), 494M(1), 496M(1) and 498M(1) exhibited a moderate antiviral effect. Two extracts 303M(2) and 475M(1) showed slight inhibition, while the remaining twelve extracts showed no meaningful antiviral effect.

### 2.4. Viral Replication Inhibition Assay

**Figure 3:**
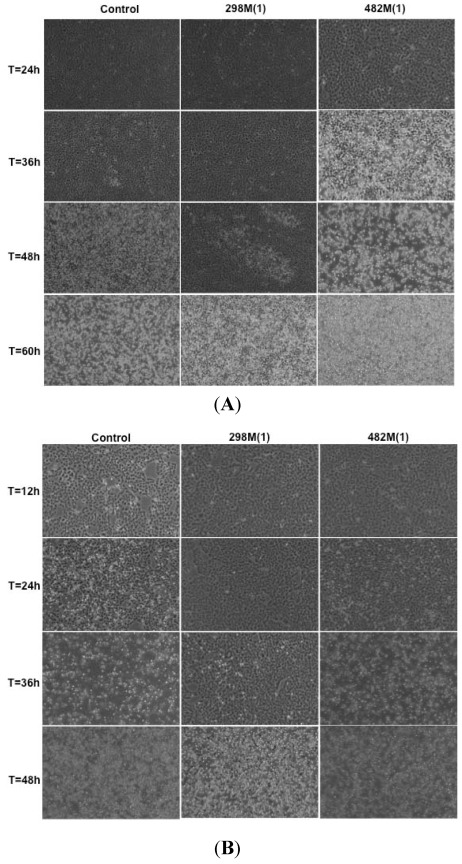
Inhibitory effect of marine microbial extracts on viral replication. (Vero cells were seeded into T-12.5 cm^2^ flasks and then infected with VSV (**A**) or poliovirus-1 (**B**) at an MOI of 0.001. Following 1 h viral adsorption, culture fluid was completely removed and infected cells were washed three times and then incubated with the medium containing selected marine extracts at subtoxic concentrations. Photomicrographs were taken to show the progression of viral-induced CPE at selected post-infection times. In addition, delayed progression of CPE in the presence of marine extracts 298M(1) was documented compared to control cells under the same experimental condition except with no marine extract.)

In addition to antiviral attachment/entry studies, selected marine extracts were tested for their potential to disrupt the viral replication process. Results obtained from this study showed only a few marine extracts were able to interfere with viral replication in different efficiency. As shown in Figure 3, extract 298M(1) showed antiviral activity against poliovirus-1 and VSV. This inhibitory effect was evidenced by delaying the appearance of viral induced-CPE for 36 h and 48 h, respectively as compared to the infected control cells. However, this antiviral effect, was not observed at 60 h after infected. Similarly, extracts 303M(1), 457P(1), 457M(1), 474M(1), 476M(1), 479M(1), 482M(1) and 496M(1) all showed some signs of against VSV replication at an early post-infection time point (24 h), however such antiviral effects vanished at 48 h post-infection. None of the other marine extracts tested showed apparent inhibitory property against the 4 selected viruses based on the measurement of viral replication and production by CPE (Figure 3). 

### 2.5. Isolation and Identification of Antiviral Components in Crude Extracts

**Figure 4:**
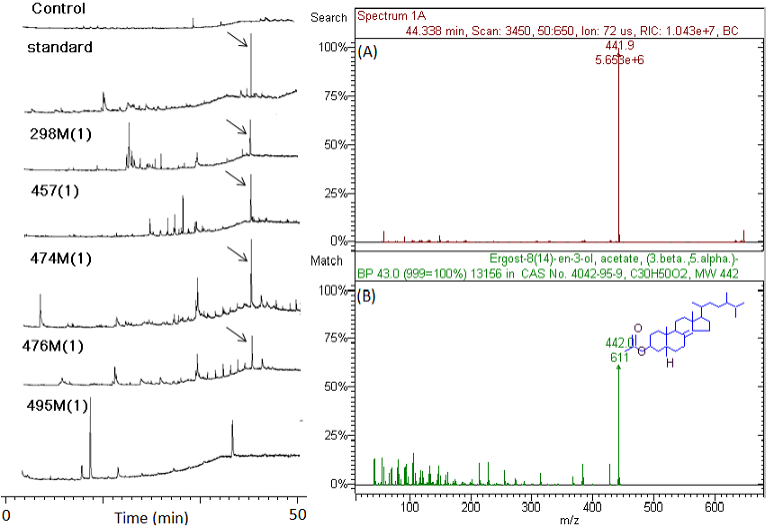
GC/ITMS chromatograms of marine microbial crude extracts including blank control, standard, 298M(1), 457P(1), 474M(1), 476M(1) and 495M(1). (**A**) Mass peak of target compound in marine microbial crude extracts; (**B**) Mass peak of standard in database of GC-ITMS workstation. Arrow point denotes target compound.

Figure 4 presents typical GC/ITMS chromatograms of marine microbial crude extracts including blank control, standard, 298P(1), 457P(1), 474M(1), 476M(1), and 495M(1). Results show that the peak of arrow point is the most commonly monitored compound in all marine microbial crude extracts with antiviral activity such as 298P(1), 457P(1), 474M(1), and 476M(1). In addition, analytical results also show that the commonly monitored peak in GC/ITMS chromatograms was included in all 4 extracts tested with antiviral activity. However, the commonly monitored peak in GC/ITMS chromatograms was not detected in 3 marine microbial crude extracts 456P(1), 495M(1), and 496P(1), which have no antiviral activity. 

Matching the mass spectra and GC retention times with its standard identified a significant compound, secosteroid. Secosteroids are highly oxidized metabolites with bond cleavage in the rings of the steroid tetracyclic nucleus. All secosteroids are grouped in accordance with their ring joined to side chain as 5,6-, 9,11-, 9,10- 8,9-, 8,14- and 13,17-secosteroids and the structures and synthetic works, where available, are reported. A review describes the isolation from marine organisms of all secosteroids reported in the literature from 1972 to 2004 and gives details on the biological activities of the isolated secosteroids (e.g., antiproliferative, antifouling, anti-inflammatory, antimicrobial, ichthyotoxic and antiviral) [10].

### 2.6. Discussion

Testing marine natural products for their therapeutic potential has been a continued focus for new drug development and such effort has led to the discovery of many important therapeutic agents. The global marine preclinical pipeline reported a total of 592 marine compounds with antitumor activity for the period of 1998–2006, and 666 additional chemicals with a variety of pharmacological activities including antibacterial, anticoagulant, anti-inflammatory, antifungal, anthelmintic, antiplatelet, antiprotozoal and antiviral activities. In addition, these chemicals also showed some effect on the cardiovascular, endocrine, immune, and nervous systems [11,12]. To extend the current effort for the discovery of new antiviral drug from marine environment, 38 newly-prepared extracts from marine microorganisms isolated from Hawaiian waters were tested for their antiviral effect against four mammalian viruses. Our screening study demonstrated that some of these extracts are highly active against the test viruses (>80% viral inactivation), particularly to enveloped viruses.

Prior to the screening of marine extracts for their antiviral activity, a set of experimental tests was carried out to determine the maximum safe dose of these extracts to be used in test cell cultures. Since these extracts were crude preparations and not purified compounds, they were tested at 100 μg/mL, the maximum test concentration according to Verkman’s drug discovery theory [13]. Any extract producing 10% or more reduction in cell viability was considered to be toxic and IC_10_ values were calculated as the subtoxic concentration in the latter experimental tests in this study.

Antiviral activity of these marine extracts was evaluated by using three different methods. First, the extracts were tested for their ability to prevent viral infection by blocking cellular receptors of permissive cells. Data obtained from viral infectivity tests showed that the pretreatment of Vero cells with these extracts resulted in no apparent reduction of viral plaque formation, suggesting these extracts have no preventive effect on initial viral infection. Similarly, screening of the marine extracts for possible disruption of viral application revealed that none of the extracts was affective. Exception for a few extracts which exhibited limited antiviral activity against VSV and poliovirus-1 during early phase of viral replication, treatment with the extracts resulted in no apparent decrease in viral production in test cells as compared to untreated control cells evaluated by the appearance and development of viral-induced CPE and the production of infectious virus particles.

Lastly, the marine extracts were tested for their ability to combat viral infection by blocking viral attachment/entry into the cells and our experimental results demonstrated that several marine extracts are highly inhibitory to all enveloped viruses tested, including VSV, HSV-1 and vaccinia virus. However, none of these extracts showed any antiviral activity against naked poliovirus-1. These findings suggest that the observed viral inactivation appears to be envelope-based but non-viral specific since VSV, HSV-1 and vaccinia are classified into three different viral families. Although little is currently known regarding the mechanism relating to the observed viral inactivation, the formation of a stable virion-complex with any compound that has special structures in these extracts may be the explanation. It is known that the occupancy or denaturation of viral surface proteins can lead to the loss of viral ability to interact with host cells. 

Because of the limited number of marine extracts tested in this study, it is impossible to claim a clear correlation between antiviral activity and the extract’s organism of origin. However, the present study showed that some extracts from marine bacterial species have antiviral potential. This finding is consistent with previous reports from several other groups indicating the virus inactivating capacity from marine bacterial species [14,15,16,17]. Current screening of natural products derived from marine microbial species has identified metabolites with significant antiviral activity, although only a few were derived from marine bacterial species [18]. This may be largely due to the limited amount of studies focused on finding bioactive compounds from a few marine bacteria, such as exopolysaccharides and macrolactin A. Undoubtedly, continuous screening of more extracts from marine bacteria will enhance present pharmaceutical development by the discovery of more potential antiviral molecules from marine bacteria. In particular, since the low abundance of the natural producers and/or the low concentrations of the compounds of interest have had a limiting effect, hampering the development of new marine drugs, and the highly complex structures of many marine metabolites also make synthetic approaches for their development economically daunting, the biomedical potential of marine bacterial agents that can be amenable to biotechnological production might overcome these problems based on the supply and sustainability of microorganisms from the sea.

This study also indicated that some extracts from marine diatoms including 457P(1) and 457M(1) (Diatom, *Amphora* sp.) are highly active against all three enveloped viruses: HSV-1, VSV and vaccinia. This result is in agreement with the findings from our previous study [19] and the report by Lee and co-workers who demonstrated the bioactive compound naviculan isolated from diatom species (*Navicula directa*) to have antiviral activities against herpes simplex viruses 1 and 2, and influenza A virus [20]. These findings together suggest the importance and essentiality of exploring additional marine diatom extracts for antiviral compounds. The diatoms represent a large and extraordinary ecologically flexible group of unicellular eukaryotic photosynthetic microalgae. The species diversity of diatoms is large, with estimated species range from 1 × 10^4^ to 2 × 10^5^ [21], which constitute one of the major components of the phytoplankton in both freshwater and marine environments [22]. However, little is currently known about bioactive substances in diatoms, as compared to other abundant aquatic microorganisms like cyanobacteria and dinoflagellates [23]. Therefore, testing and evaluation of extracts and compounds from marine diatom species will become a major research focus on discovering marine antiviral drugs and other therapeutic remedies for humans in future.

It is notable that several extracts showed antiviral activity against VSV, HSV-1 and vaccinia, but the antiviral capacity among these three enveloped viruses is quite different. In particular, extracts 298M(1), 303M(2), 496M(1) and 62P(1) exhibited much more potent antiviral effects or viral inactivation activity against VSV as compared to the other two viruses. This selective anti-VSV tendency documented in the previous report [18] may be due to the nature of the envelope proteins of rhabdoviruses. VSV is a virus member belonging to *Rhabdoviridae* family and comprises a helical ribonucleocapsid surrounded by a lipid bilayer covered by trimers of a single type of an integral glycoprotein, named G protein. Cell recognition and fusion are mediated by VSV surface glycoprotein G and then viral entry thorough endocytosis followed by low-pH-induced membrane fusion [24]. On the other hand, extracts 457P(1), 457M(1) and 482M(1) all showed the highly potential antiviral effect across all tested enveloped viruses, this may suggest that the glycoproteins of these enveloped viruses are the viral component responsible for the reaction with any fractions of these extracts. Future study is necessary in order to confirm and fully understand this observation.

To explore and identify any component of marine extracts with antiviral potential responsible for the observed antiviral effect, representative extracts were further extracted and then analyzed by GC-ITMS. A compound, 5α(*H*),17α(*H*),(20*R*)-β-Acetoxyergost-8(14)-ene was putatively identified by matching its mass spectra and GC retention times with its standard in almost all microbial extracts tested with antiviral activity in the present study. 5α(*H*),17α(*H*),(20*R*)-β-Acetoxyergost-8(14)-ene belongs to secosteroids, highly oxidized metabolites with bond cleavage in the rings of the steroid tetracyclic nucleus. The first marine secosteroid to be described was discovered in the gorgonian *Pseudopterogorgia americana* in 1972 [25]. The majority of secosteroids has been isolated from sponges, gorgonians, soft corals and from the ascidian *Aplidium conicum *[10]. The characterization of secosteroids was made possible by the continuing improvement of the separation techniques and the introduction of new identification methods. Currently we cannot explain the biological function and the mechanism of the biosynthesis of these novel modified steroids. However, secosteroids have been reported to have diverse biological activities, e.g., antiproliferative, antifouling, anti-inflammatory, antimicrobial, ichthyotoxic and antiviral [10]. 

In summary, screening of 38 extracts from marine bacteria, diatoms and other microorganisms allowed the identification of several extracts with potent antiviral activity, particularly on viral inactivation for enveloped viruses. Among the tested samples, extracts 457M(1), 457P(1) and 482M(1) revealed to have antiviral potential with a broad-spectrum of viruses. However, the observed inhibition does not seem sufficient to suggest the application of these microbial extracts as treatment of an established viral infection. Since these extracts have the potential to be used as prophylactic agents to prevent viral infection, as well as preventing viral spread as evidenced by the high level of viral inactivation without cytotoxicity, it would be important and of interest for future studies to focus on the isolation of the active fractions and purified compounds from these extracts. Identification of active individual chemical components of the extracts and study of their chemical properties against specific viral genomic or proteomic components will lead to the understanding of direct anti-viral mechanisms and facilitate their development as new antiviral drugs.

## 3. Experimental Section

### 3.1. Cell Culture and Media

Green Africa monkey kidney (Vero) cells (ATCC, Manassas, VA, USA, Cat, No. CCL-81TM) were grown with Dulbecco’s Modified Eagle Media (DMEM) (Sigma-Aldrich, St. Louis, MO, USA) supplemented with 10% heat-inactivated bovine calf serum (BCS) (Hyclone, Logan, UT, USA) and 1% GPS solution containing 4 mM L-glutamine (Sigma-Aldrich), 100 U/mL penicillin and 100 μg/mL streptomycin sulfate at 37 °C with humidified 5.0% CO_2_. 

### 3.2. Virus Stock Preparation

The viral isolates (Table 1), their replication and purification and quantitative infection assays have been established and routinely used in the laboratory, University of Hawaii at Manoa [26]. These model viruses were propagated and quantified. Briefly, Vero cells were grown and seeded into TC-75 cm^2^ flasks, as described above, so that an approximately 90% cell monolayer formed in 24 h. All medium was removed from the flask and the cells were infected with 250 μL previously prepared virus stock mixed together with 2 mL of serum-free medium. The flasks were incubated at 37 °C for 1 h and then unadsorbed virus was removed by rinsing cell the monolayer twice with serum-free medium, and then 10 mL/flask virus replication medium containing 5% BCS was added. The flasks were then incubated at 37 °C for viral infection and production. Flasks showing approximately 90% CPE evidenced by visual appearance of rounding of cells, loss of contact inhibition and cell death were harvested and stored at −80 °C for 24 h. Following two cycles of freeze-thawing, the medium was collected from the flasks and centrifuged at 1000 rpm for 5 min to remove cellular debris. Supernatant was then collected and aliquots of 0.5 mL/tube were stored long-term at −80 °C or short-term at −20 °C. 

### 3.3. Marine Microbial Isolates

Thirty-eight marine microbial isolates were obtained from Thomas Hemscheidt’s laboratory at the University of Hawaii at Manoa. These microbial isolates were collected from sites around the Hawaiian coastal waters. Briefly, pure cultures were established from these samples, identified taxonomically by means of PCR amplification and subsequent sequencing of small subunit ribosomal RNA (ssrRNA) genes, and entered into the Center for Marine Microbial Ecology and Diversity (CMMED) culture collection, and cryopreserved in quintuplicate.

### 3.4. Marine Microbial Extracts

To prepare extracts for evaluation of antiviral activity, 2 L cultures were grown subsequent to inoculation with a cryopreserved sample. Prior to harvest of the culture, a subsample is subjected amplification and sequencing of ssrRNA genes to confirm taxonomic identity. Cultures were harvested by centrifugation at 5000 g for 15 min. Supernatant medium was extracted with ethyl acetate; the ethyl acetate fraction was then evaporated to dryness; the residue was then resuspended in a small volume of ethyl acetate, and transferred to a liquid scintillation vial. Ethyl acetate was then removed under vacuum, and the vial weighed to determine the amount of material recovered. The cell pellet remaining after centrifugation was extracted in 2:1 methylene chloride: 2-propanol. The resulting organic fraction was evaporated to dryness, the residue re-suspended in a small volume of extraction solvent, and transferred to a liquid scintillation vial. The solvent was removed under vacuum, and the vial weighed to determine the amount of material recovered. The samples were then dissolved in DMSO at a concentration of 100 mg/mL and then used for screening.

### 3.5. Cytotoxicity Assay by Cell Viability

Cells at exponential growth phase were harvested by trypsin-versene solution, and seeded at 1 × 10^4^ cells per well in 96-well plates. Following 24 h incubation at 37 °C, a confluent cell monolayer was confirmed and cell media was removed. Extracts were serially diluted with the culture medium supplemented with 10% serum to reach the concentrations of 100, 50, 25, 12.5 and 6.25 μg/mL. Control dilution of DMSO at 0.1% was also included. An aliquot of 200 μL/well of each diluted extract or DMSO was added to the plates in 4 replicates. The plates were incubated for 2 day at 37 °C. A methylthiazol tetrazolium (MTT) assay commonly used for cell proliferation was adopted to test cell viability [27]. 

### 3.6. Viral Plaque Reduction Assay

To establish quantitative measure method, cells at exponential growth phase were harvested and seeded at 4 × 10^5^ cells/well of 6-well plates for test viruses. This cell seeding density would allow the formation of an approximately 90% monolayer within 24 h. Once a confluent cell monolayer was formed, cell growth medium was aspirated from the wells. Meanwhile, serial 10-fold dilutions of a virus stock were prepared and added to the plates at 200 μL/well with 5 wells/viral dilution. Plates were incubated for 1 h at 37 °C, with gentle shaking back and forth and side-to-side every 15 min to enhance even virus distribution. Residual virus was completely removed from each well and 2 mL/well of a 0.75% (w/v) methylcellulose overlay medium containing 2% serum was added. Plates were then incubated for 3–4 days to allow viral plaque development. Viral plaques were visualized by the addition of 1 mL/well of crystal violet staining solution for at least 3 h [28] at room temperature followed by vigorous washing with tap water. Plaques were counted visually and the viral titer calculated as follows: Virus Titer (PFU/mL) = [# plaques counted × dilution factor/amount of viral inoculum used (0.2 mL)].

### 3.7. Viral Adsorption Assay–Cell Pretreatment

Cells at an exponential growth phase were harvested and seeded at 2 × 10^5^ cells per well in a 12-well plate to allow the formation of an approximately 90% cell monolayer in 24 h. Microbial extracts were diluted with serum-free medium to sub-toxic concentrations, as determined by the cytotoxicity test. The medium was then aspirated from the well and an aliquot of 100 μL extract was added into each well. After 1 h incubation, the extract was aspirated from the well and the cell monolayers were rinsed twice with DPBS (Sigma-Aldrich, St. Louis, MO, USA). A prepared viral aliquot containing 25–50 PFU/100 μL was added per well and the plates were incubated at 37 °C for 1 h to allow viral adsorption. Extracts producing a reduction in plaque formation were considered for further characterization. Antiviral effects of each extract were categorized as having no meaningful inhibition (<20%), slight inhibition (≥20% and <50%), moderate inhibition (≥50% and <80%), or high inhibition (≥80%). All experiments were conducted in triplicate.

### 3.8. Viral Adsorption Assay–Virus Pretreatment

Cells at an exponential growth phase were harvested and seeded at 2 × 10^5^ cells per well into a 12-well plate to allow the formation of an approximately 90% cell monolayer within 24 h. Microbial crude extracts were diluted with serum-free medium to twice the sub-toxic concentrations (e.g., 200 μg/mL for those found to be nontoxic at 100 μg/mL) as determined by the cytotoxicity tests. Viruses were diluted in serum-free medium to optimum concentrations of 50–100 PFU/well as determined by previous tests. Then, 100 μL of each extract at twice the maximum sub-toxic concentration was mixed with an equal volume of the virus dilution. Positive controls were made by mixing 100 μL of virus dilution with 100 μL of serum-free medium with 0.2% DMSO, in order to yield a final DMSO concentration of 0.1%. These virus-extract mixtures were pre-incubated for 1 h at room temperature before viral infectivity test under the optimized plaque assay conditions. Extracts leading to a reduction in plaque formation were considered for further characterization. Antiviral effect of each extract was categorized as having no meaningful inhibition (<20%), slight inhibition (≥20% and <50%), moderate inhibition (≥50% and <80%), or high inhibition (≥80%). All experiments were conducted in triplicate.

### 3.9. Viral Replication Inhibition Assay

Test cells were seeded at 4 × 10^5^ cells into a TC-12.5 cm^2^ flask (BD Falcon, San Jose, CA, USA) to allow the formation of an approximately 90% cell monolayer within 24 h. After the medium was completely aspirated from the flask, the cell monolayer was briefly washed twice with DPBS and then infected with selected test virus at a multiplicity of infection (MOI) of 0.001. After 1 h viral adsorption, the virus was completely removed and the flask was washed twice with DPBS. Infected cultures were then incubated with medium containing 2% BCS and diluted marine extracts at their safe and effective concentrations, as determined by the cytotoxicity tests. Duplicate flasks were used per test extract and these cultures were allowed to incubate for 3 days. Under the same condition, Vero cells were infected with test virus in the absence of any marine extract as a positive control. Development of viral induced CPE was documented by taking photomicrographs every 12 h using an inverted phase-contrast microscope equipped with a digital camera (Nikon Eclipse TE2000-U). To track viral production, 200 μL aliquots of the medium were sampled from each flask every 12 h and stored at −20 °C until the end of the experiment. The viral titers of these samples were later determined by the plaque assay described above. Test extracts that produced a visually noticeable reduction in CPE and reduction in viral titers were further characterized.

### 3.10. Marine Microbial Extract Clean-Up and Fractionation

Representative crude marine microbial extracts were extracted with a mixture of acetone and ethyl acetate (1:1, v/v) under sonication for 30 min. After the extract was dried with 10 g of anhydrous Na_2_SO_4_, it was reduced in volume to approximately 0.5 mL using a rotary evaporator and purified on an aluminum/silica column (10 cm × 8 mm i.d.). The column was packed, from the bottom to top, with neutral alumina (1.5 cm, 3% deactivated), neutral silica gel (2 cm, 3% deactivated) and anhydrous Na_2_SO_4_ (1 cm). The column was eluted with 10 mL acetone and ethyl acetate (1:1) to yield the target fraction. The fraction was concentrated to 20 L under a gentle stream of high purity nitrogen gas after 20 L of dodecane was added as the trapping solvent prior to gas chromatography-ion trap mass spectrometry analysis (GC-ITMS).

### 3.11. GC-ITMS Analysis

Marine microbial crude extracts were analyzed on a Varian 3800 GC and Saturn 2000 ITMS system (Varian, Walnut Creek, CA, USA) after clean-up and fractionation. An aliquot of 2.0 µL of sample was injected in splitless mode with an AS8400 autosampler. The purge valve was activated 2 min after the sample injection. The column flow rate was 2 mL/min (helium). The injector and ion trap temperatures were 280 °C and 230 °C, respectively. A DB-5MS capillary column (30 m × 0.25 mm i.d., 0.25 μm film thickness) was used for separation of microbial crude extracts. The oven temperature started at 90 °C for 1 min, increased to 290 °C at a rate of 4 °C min^−1^, and then held at 290 °C for 10 min. The ITMS was operated under full ion scan mode.

### 3.12. Data Analysis

The analysis of variance (ANOVA) tests were first performed for each sample to test the differences of variability among different concentrations. For those samples with no significant differences, the 100 μg/mL is used as the testing concentration for future tests. For samples with significant differences among different concentrations, an IC_10 _concentration was calculated based on the probit curve of the variability. All statistical analyses were performed using SPSS 16.0 software [9] with significance level controlled at 5%.

## 4. Conclusions

The microbial extracts showed broad-spectrum antiviral potencies as prophylactic agents to prevent enveloped viruses infection, as evidenced by their high inhibition against all enveloped viruses tested. 5α(*H*),17α(*H*),(20*R*)-β-Acetoxyergost-8(14)-ene in the marine microbial extracts was putatively identified and confirmed to be associated with antiviral activity.
